# Correlates and cascade of HIV care in patients with psychiatric disorders in the Eastern Cape province, South Africa

**DOI:** 10.4102/sajpsychiatry.v28i0.1753

**Published:** 2022-02-15

**Authors:** Adila Aboobaker, Zukiswa Zingela, Oladele V. Adeniyi

**Affiliations:** 1 Department of Psychiatry and Behavioural Sciences, Faculty of Health Sciences, Walter Sisulu University, East London, South Africa; 2 Department of Psychiatry, Cecilia Makiwane Hospital, Mdanstane, East London, South Africa; 3 Department of Psychiatry and Behavioural Sciences, Faculty of Health Sciences, Walter Sisulu University, Mthatha, South Africa; 4 Department of Psychiatry, Mthatha Hospital Complex, Mthatha, South Africa; 5 Department of Family Medicine and Rural Health, Faculty of Health Sciences, Walter Sisulu University, East London, South Africa; 6 Department of Family Medicine, Cecilia Makiwane Hospital, East London, South Africa

**Keywords:** cascade of care, continuum of care, HIV prevalence, linkage to care, psychiatric disorders, South Africa, virologic suppression

## Abstract

**Background:**

The cascade of human immunodeficiency virus (HIV) care in patients with psychiatric disorders is poorly understood.

**Aim:**

This study determined the prevalence of HIV and described its cascade of care among patients with psychiatric disorders in the Eastern Cape province, South Africa. The study also examined the correlates of HIV comorbidity with psychiatric disorders in the cohort.

**Methods:**

In this cross-sectional study, a total of 368 individuals attending the Psychiatric Outpatients’ Department of Cecilia Makiwane Hospital in Eastern Cape were interviewed with a structured questionnaire. Relevant items on demographics and clinical information were extracted from the medical records. Virologic suppression was defined as viral load < 1000 RNA copies/mL.

**Results:**

The HIV prevalence after the intervention was 18.8% and a significant proportion of participants already knew their status (*n* = 320; 87.0%). Linkage to care and antiretroviral therapy initiation occurred in 61 participants, of those diagnosed with HIV (88.4%), with 84.1% being eligible for viral load monitoring (*n* = 58) and 53.4% having achieved virologic suppression. Being female (AOR = 5.48; 95% CI 2.61–11.51) and black (adjusted odds ratio [AOR] = 3.85; 95% confidence interval [CI] 1.06–14.03) were independent predictors of HIV comorbidity in individuals living with psychiatric disorders.

**Conclusion:**

This study found a moderately high prevalence (close to 19%) of HIV in individuals with psychiatric disorders, with a significant correlation with being female and being black people. This study also found a significant gap in the linkage to antiretroviral therapy (ART) initiation and a low rate of virologic suppression of 53.4%. Clinicians, therefore, should monitor and provide interventions for patients with concomitant HIV infection along this cascade of care.

## Introduction

The cascade of human immunodeficiency virus (HIV) care is defined as the diagnosis of HIV, linkage to care and initiation of antiretroviral therapy (ART), retention in care, and virologic suppression.^1^ Globally, 37.4 million people were living with HIV by the end of 2018; 79% of them were aware of their serostatus. With increasing access to ART worldwide, 24.5 million people were already on ART (62% of all people living with HIV [PLWHIV]) by June 2019 and 52% had achieved virologic suppression at the end of 2018.^2^ In South Africa, 7.7 million people were living with HIV by the end of 2018; 90% knew their status, 62% were already initiated on ART and 54% had achieved virologic suppression.^2^ While the HIV care cascade has been reported for the general population, it is poorly understood among psychiatric patients with HIV infection.

Organisations prioritise children, pregnant women, sex workers, men having sex with men, transgender people, and people injecting drugs as the key populations for HIV testing.^3,4^ Yet people with psychiatric disorders seem to be a neglected population, even though their vulnerabilities to HIV are well documented.^5,6,7,8,9,10^ This is evidenced by the silent treatment meted out to people with psychiatric disorders in many national and international guidelines and policies.^3,4^ Many authors have reported on the mental health of PLWHIV,^11,12,13^ yet there is a lack of information regarding people with psychiatric disorders living with HIV.

It is also worth noting that one systematic review and meta-analysis carried out by Hughes et al.,^10^ found that the prevalence of HIV among people with mental illnesses is higher than the general population in countries with low HIV prevalence and on par in countries with high HIV prevalence. Given that the comorbid diagnosis of HIV and psychiatric disorder tends to attract a stigma,^14,15,16^ people with these comorbidities need special care. Robust evidence to guide HIV care for people with psychiatric disorders is lacking and controversies exist surrounding the consent for testing in this population. Some clinicians insist on receiving informed consent to perform HIV testing, yet many people with psychiatric disorders are unable to give informed consent because of the severity of their condition.^17,18^ As such, some patients are not tested for HIV, nor initiated on ART, thus leading to the deterioration and possible fatal outcomes. National recommendations on the treatment of all individuals living with HIV is contingent on HIV testing and linkage to care.^4^

The bidirectional relationship between HIV and psychiatric disorders has been extensively documented in the literature.^6,7,8,9,10^ People living with HIV have an increased risk of having a psychiatric disorder. Similarly, people living with a psychiatric disorder have an increased risk of acquiring HIV.^6,7,8,9,10^ Given that the viral load is the most important determinant of progression of HIV disease,^19^ evidence suggest that the comorbidity of psychiatric disorder is associated with lower odds of achieving virologic suppression.^20^

A Swedish study showed that adults with psychiatric disorders have an almost three-fold increased risk of being infected with HIV when compared to the general population.^21^ Similarly, a report by Singh also showed that individuals with psychiatric disorders are three times more likely to be infected with HIV compared to the general population in South Africa.^18^ However, a meta-analysis conducted by Breuer et al. reported that PLWHIV are twice more likely to develop depression in comparison to the general population.^22^ The results of these studies indicate a bidirectional relationship that exists between psychiatric disorders and HIV. Adams et al. reported depression as the predominant comorbid psychiatric disorder (47%) among PLWHIV in London,^11^ while 17% had adjustment disorders, 15% had anxiety disorders, and 15% were living with substance abuse. Similarly, Chibanda et al., reported that the three most prevalent psychiatric disorders in PLWHIV in low- and middle-income countries, including South Africa, were depression, alcohol use disorders, and neurocognitive disorders.^12^

The prevalence of HIV ranges from 13% in an inpatient setting in the Western Cape to 50% among first-episode psychosis presentations in KwaZulu-Natal (KZN) or 29.01% among general psychiatric inpatients in KZN among people with psychiatric disorders in South Africa.^18,23,24^ Studies have reported that women with psychiatric disorders were twice as likely to get infected with HIV than their male counterparts.^5,18^ Poor adherence to ART, and the resulting increased viral load because of virologic failure, immunological failure, and clinical failure have been documented in individuals with the comorbidity of HIV and psychiatric disorders.^25,26,27,28,29^ A six-fold higher viral load was found in individuals with severe psychiatric disorders and HIV in comparison to individuals living with HIV and no psychiatric disorder.^26^

There is a dearth of information on the comorbidity of HIV and psychiatric disorders in the Eastern Cape province, one of the poorest provinces in the country. Uys found an HIV prevalence of 13% among female patients admitted to a regional hospital (the same site for the current study) in the Buffalo City metropolitan municipality (BCMM) in the Eastern Cape province.^30^ A predominant proportion (53%) of the study participants developed a psychiatric disorder after receiving an HIV diagnosis, while the rest of the patients had a pre-existing primary psychiatric disorder. It should be noted that the small sample size and the sampling from female inpatients did not allow for a broader understanding of the comorbidity of HIV and psychiatric disorders in this population. Therefore, this study aimed to bridge this gap and, to provide relevant epidemiological data to guide the development of integrated psychiatric care services in this region of the province.

## Aim

This study aimed to determine the prevalence of HIV and quantify the HIV care cascade in individuals attending psychiatric services. The study also assessed the independent risk factors of the comorbidity of HIV and psychiatric disorders in the cohort.

## Methodology

### Study design and setting

This descriptive, cross-sectional study was conducted at the Cecilia Makiwane Hospital’s (CMH) outpatients’ department (OPD), in the Eastern Cape province, between October and November 2018. This is an academic department affiliated to the Walter Sisulu University (WSU) for the training of undergraduate and postgraduate students in psychiatry. This hospital provides psychiatric services to the entire central region of the Eastern Cape Province, serving a combined population of 1 674 637 residents in the BCMM and Amathole District.^31^

### Study population

The racial distribution for the two districts includes black people (97.24% in Amathole and 85.11% in BCMM), white people (1.0% in Amathole and 7.71% in BCMM), mixed race (1.47% in Amathole and 6.0% in BCMM), Indian/Asian (0.13% in Amathole and 0.83% in BCMM), and ‘Other’ race (0.16% in Amathole and 0.33% in BCMM).^31^

The unit accepts referrals for all categories of mental disorders for assessment and treatment. All patients attending the outpatient clinic were eligible for recruitment into the study. Ideally, all patients referred to the study setting should have undergone a full medical work-up (including HIV test) prior to referral.

### Inclusion and exclusion criteria

Patients who were at least 18 years old and already in care for psychiatric disorders at the psychiatric OPD of the hospital were recruited for this study. Acutely ill, violent, and critically unstable patients who needed either emergency or inpatient care were excluded from the study.

### Sample size estimation

To be able to make the study findings more generalisable to the study population, a sample size of 368 participants was estimated using the following formula for cross-sectional study:

$$\left\{\text{N}={\left({\text{Z}}_{1}-\alpha \right)}^{2}\times \text{P}(1-\text{P})/{\text{D}}^{2}\right\}$$ [Eqn 1]

Where Z is the confidence level, D is the margin of error and P is the expected proportion of patients with an awareness of HIV status.

**FIGURE 1:**
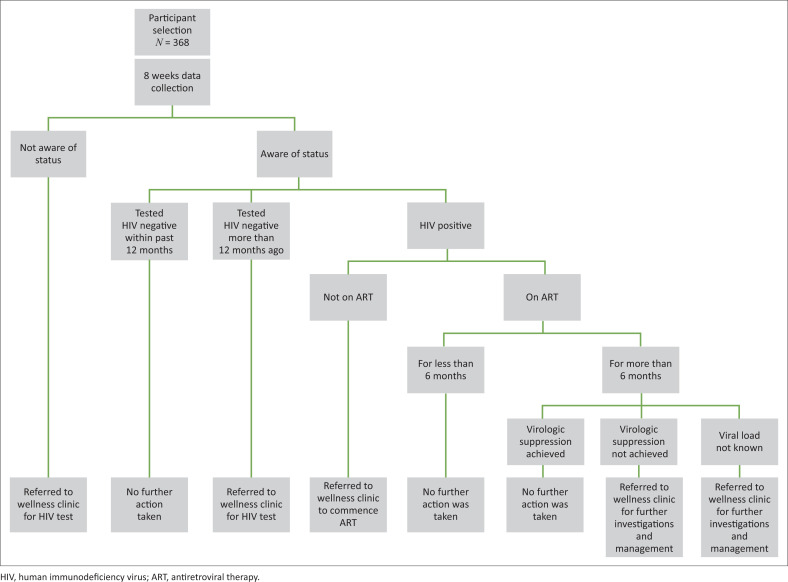
Study procedure and interventions.

Previous study in the same setting reported 71.1% awareness of HIV status among patients attending emergency department.^32^ Hence, P was set at 70% and D at 0.05. The calculation was performed at 95% confidence level (CI). The participants were selected consecutively on each day until the sample size was attained (see Figure 1).

### Data collection

The participants were recruited after their consultation with the attending psychiatric clinician. The clinician offered a consent form, completed a face-to-face interview, including a review of medical records, and completed a structured questionnaire for the study. This questionnaire was self-designed by the principal researcher and was piloted with 22 participants. Feedback from the participants and clinicians in the study setting was used to improve the instrument. Results of the pilot study were not included in this study.

Data on demographic information (age, gender, marital status, race, education level, and employment status), mental illness, most recent HIV diagnosis, treatment for HIV, and recent viral load were collected. Age was categorised into the following year groups: 18–24, 25–34, 35–49, and greater than or equal to 50. Marital status was categorised as follows: single, married, divorced, and widowed. The racial distribution was documented as black people, white people, and others (mixed race and Asian). The attained level of education was categorised as: primary (grade 1–6), secondary (grade 7–12), and tertiary (post-secondary). The underlying psychiatric disorders recorded on medical records were categorised as: primary depressive, primary psychotic, primary bipolar, primary anxiety, other primary psychiatric, depressive disorder as a result of HIV, psychotic disorder as a result of HIV, a bipolar disorder as a result of HIV, psychotic disorder as a result of another medical condition (AMC) other than HIV, a bipolar disorder as a result of AMC other than HIV, psychotic disorder as a result of substance abuse and depressive disorder as a result of substance abuse.

## Main outcome measures

### HIV prevalence

Estimated after testing all the participants with unknown or negative status tested more than 12 months before the study, and adding that to the number of patients with known HIV positive status.

### HIV status awareness

Confirmed through participants’ self-reporting or by participants’.

### Linkage to care and ART initiation

The participants’ medical records provided evidence for the initiation of ART.

### Virologic suppression

It was defined as the most recent viral load less than 1000 ribonucleic acid (RNA) copies/mL. The participants’ medical records supplied this information. Viral load results were recorded at 6 months if newly initiated on ART, and at 12 months if the participant had been on ART for more than a year, following the national guideline.^4^

### Intervention

Although this was an observational study, the research team assisted each participant when necessary to bridge any identified gaps in their care for both HIV and psychiatric disorders in the cohort. All participants who had unknown or negative HIV status in the preceding 12 months were re-tested following the National Department of Health’s Guidelines for HIV Testing.^4^ The results of those who were re-tested were included in the final analysis. Also, participants who had HIV diagnosis and who were not yet on ART were referred to the CMH Wellness Clinic for the initiation of treatment. Those who were already on ART without any recent viral load were referred for viral load assessment.

### Ethical considerations

The WSU Human Research and Ethics Committee approved the study (Reference number: 092/2017). The Eastern Cape province Department of Health (Reference number: EC_201806_007) also approved the study and the Clinical Governance of CMH granted permission for the implementation of the study. Each participant, or the legal guardian, gave written informed consent before participation. The participants’ anonymity, privacy and confidentiality were safeguarded through password protection of access to study data and the interviews were conducted in a private space.

## Statistical analysis

Data were entered into Microsoft Excel and exported to the Statistical Package for Social Sciences (SPSS) Version 25.0 for Windows (SPSS Inc, Chicago, Illinois, USA). To ensure accuracy, data cross-checking by the researcher and an assistant was conducted before analysis. The data were expressed as mean values ±standard deviation (SD) for continuous variables. Counts (frequencies = *n*) and proportions (%) were reported for categorical variables. The sociodemographic characteristics of the participants, the prevalence of HIV, and the proportions of participants along the HIV cascade of care were summarised using simple descriptive statistics. Bivariate and multivariate logistic regression (model) analyses were performed after adjusting for confounding factors, to determine the significant risk factors of comorbidity of HIV and psychiatric disorders in the cohort. A *p*-value of less than 0.05 was considered statistically significant.

## Results

### Demographic and clinical characteristics of the participants

Of the total participants (*n* = 368), the male to female distribution (50.8% versus 49.2%) was roughly equal. The highest proportion of the participants were at least 50 years old (*n* = 144; 39.1%). The majority of the participants were single (72.0%), black people (89.1%), had attained primary education (63.0%), and were unemployed (91.0%). Most of the participants (*n* = 272, 73.9%) were diagnosed with a primary psychiatric disorder, while 34 (9.2%) participants were diagnosed with a psychiatric disorder secondary to HIV infection. A total of 43 participants had a psychiatric disorder secondary to AMC (such as epilepsy), and 19 participants (5.2%) had psychiatric disorder because of substance abuse (Table 1).

**TABLE 1:** Characteristics of the participants.

Characteristics	Frequency (*n*)	%
**Gender**		
Male	187	50.8
Female	181	49.2
**Age (years)**		
18–24	28	7.6
25–34	108	29.4
35–49	88	23.9
≥ 50	144	39.1
**Marital status**		
Single	265	72.0
Married	72	19.6
Divorced	16	4.3
Widow	15	4.1
**Race**		
Black	328	89.1
White	28	7.6
Others	12	3.3
**Employment status**		
Employed	33	9.0
Unemployed	335	91.0
**Educational level**		
Tertiary	36	9.8
Secondary	100	27.2
Primary	232	63
Illiterate	0	0
**Underlying psychiatric disorder**		
Primary depressive disorder	52	14.1
Primary psychotic disorder	152	41.3
Primary bipolar disorder	62	16.8
Primary anxiety disorder	4	1.1
Other primary psychiatric disorder	2	0.5
Depressive disorder as a result of HIV	9	2.4
Psychotic disorder as a result of HIV	21	5.7
Bipolar disorder as a result of HIV	4	1.1
Psychotic disorder as a result of AMC	42	11.4
Bipolar disorder as a result of AMC	1	0.3
Psychotic disorder as a result of substance abuse	18	4.9
Depressive disorder as a result of substance abuse	1	0.3

HIV, human immunodeficiency virus; AMC, another medical condition.

### Prevalence and awareness of HIV serostatus

The prevalence of HIV in the cohort (*n* = 368) was 18.8% (*n* = 69). The majority of the participants (*n* = 320, 87%) were already aware of their HIV serostatus. Among those who were unaware (*n* = 48) of their HIV status, all tested negative following the provider-initiated counselling and testing that was offered during the study.

### Types of psychiatric disorders associated with HIV status

Of the 69 participants with comorbid diagnosis of HIV and psychiatric disorder, primary psychiatric disorders accounted for 47.8% (*n* = 33), psychiatric disorders secondary to HIV accounted for 49.3% (*n* = 34) and psychiatric disorders because of AMC accounted for the remaining 2.9% (*n* = 2) (Table 2).

**TABLE 2:** Types of psychiatric disorders associated with HIV co-morbidity.

Mental illness diagnoses	HIV negative		HIV positive		Total

	*n*	%	*n*	%	
Primary depressive disorder	47	15.7	5	7.2	52
Primary psychotic disorder	142	47.5	10	14.5	152
Primary bipolar disorder	46	15.4	16	23.2	62
Primary anxiety disorder	2	0.7	2	2.9	4
Other primary psychiatric disorder	2	0.7	0	0.0	2
Depressive disorder as a result of HIV	0	0.0	9	13.0	9
Psychotic disorder as a result of HIV	0	0.0	21	30.4	21
Bipolar disorder as a result of HIV	0	0.0	4	5.8	4
Psychotic disorder as a result of AMC	40	13.4	2	2.9	42
Bipolar disorder as a result of AMC	1	0.3	0	0.0	1
Psychotic disorder as a result of substance abuse	18	6.0	0	0.0	18
Depressive disorder as a result of substance abuse	1	0.3	0	0.0	1

HIV, human immunodeficiency virus; AMC, another medical condition.

### Linkage to care and ART initiation

**FIGURE 2:**
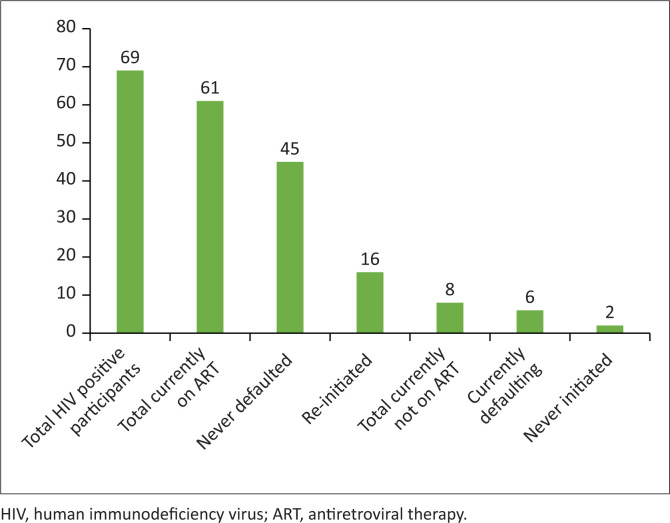
Linkage to care and ART initiation.

Of the 69 participants who were HIV positive, the majority (*n* = 61; 88.4%) were on ART at the time of the study. Of those on ART, 16 (26.0%) participants had a history of non-adherence to ART and were re-initiated at the local clinic (before the study). Six (8.7%) participants admitted to not taking ART daily in the week before the study. Eight (11.6%) participants were not on ART at the time of the study, six (8.7%) of them were non-adherent to g ART at the time of the study and two (2.9%) were never initiated. All the eight participants who were not on ART at the time of the study were referred for initiation (Figure 2).

### Virologic suppression

Of those on ART (*n* = 61) at the time of the study, the virologic suppression rate in participants who were eligible for viral load monitoring (*n* = 58) was 53.4% (*n* = 31), and 24 participants had virological failure while 3 had no viral load results.

### HIV cascade of care in the cohort

**FIGURE 3:**
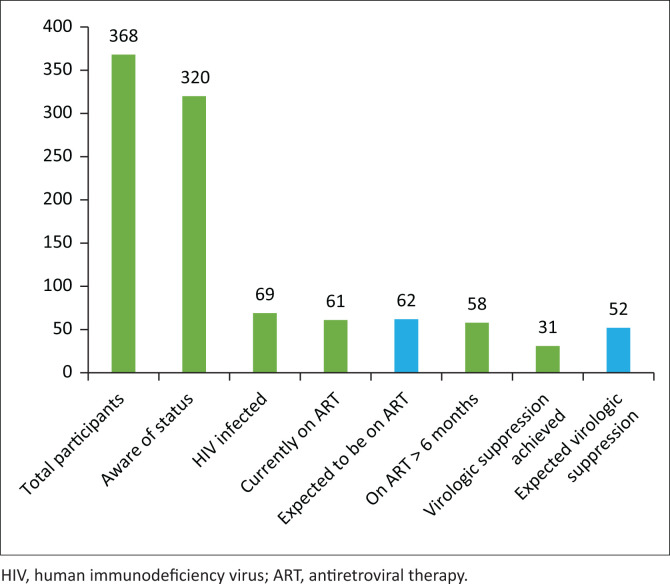
HIV cascade of care.

Figure 3 provides a graphic summary of the cascade of care for HIV in this cohort and the drop-off (blue bars) at each stage of the cascade. In keeping with the Joint United Nations Programme on HIV/AIDS (UNAIDS) 90:90:90 target, 62 (90%) participants are expected to have been on ART and not 61, as there are 69 HIV positive participants. Also, 52 (90%) of the 58 HIV positive participants on more than 6 months of ART should have attained virologic suppression, and not the low number of 31 (53.4%) participants.

### Factors associated with comorbid HIV and psychiatric disorders

In the bivariate analysis, the female gender was significantly associated with concurrent HIV and psychiatric disorder. Although the co-occurrence of HIV and psychiatric disorder was highest among the participants who were single, black, had primary education, and were unemployed, this association was not statistically significant (Table 3).

**TABLE 3:** Bivariate analysis of factors associated with HIV co-morbidity.

Variables	HIV negative		HIV positive		*p*
	*n*	%	*n*	%	
**Gender**					
Male	173	57.9	14	20.3	< 0.001
Female	126	42.1	55	79.7	-
**Age (years)**					
18–24	25	8.4	3	4.3	0.113
25–34	70	23.4	18	26.1	-
35–49	110	36.8	34	49.3	-
≥ 50	94	31.4	14	20.3	-
**Marital status**					
Single	-	-	49	71.0	0.734
Married	-	-	16	23.2	-
Divorced	-	-	2	2.9	-
Widow	-	-	2	2.9	-
**Race**					
Black	262	87.6	66	95.7	0.154
White	26	8.7	2	2.9	-
Other	11	3.7	1	1.4	-
**Education**					
Primary	189	63.2	43	62.3	0.848
Secondary	82	27.4	18	26.1	-
Tertiary	28	9.4	8	11.6	-
**Employment**					
Unemployed	275	92.0	60	87.0	0.141
Employed	24	8.0	9	13.0	-
**Living arrangement**					
Lives alone	15	5.0	5	7.2	0.470
Lives with family	275	92.0	62	89.9	-
Lives with friends	4	1.3	2	2.9	-
Lives with helper	5	1.7	0	0.0	-

#### Multivariate analysis of HIV comorbidity in this cohort

In the crude and adjusted multivariate logistic regression analysis (Table 4), being female was independently and significantly associated with HIV comorbidity while other demographic characteristics were not significant. Women were five times more likely to have HIV and mental illness comorbidity compared to their male counterparts. The magnitude and direction of association remained after adjusting for other variables. Race was not significantly associated with HIV comorbidity in the crude model. After adjusting for other variables such as age, marital status, level of education, and employment status, there was a four-fold increase in the likelihood of black participants to have HIV comorbidity with mental illness.

**TABLE 4:** Binary logistic regression showing correlates of HIV co-morbidity.

Variables	UOR (95% CI)		*p*	AOR (95% CI)		*p*
	UOR	95% CI		AQR	95%CI	
**Gender**						
Female	5.39	2.87–10.13	<0.001	5.48	2.61–11.51	<0.001
Male (ref)	1	-	-	1	-	-
**Age (years)**						
35 and above	1.06	0.60–1.88	0.829	0.79	0.41–1.51	0.472
18–34 (ref)	1	-	-	1	-	-
**Marital status**						
Never married	1.06	0.60–1.89	0.838	1.31	0.68–2.53	0.421
Never married (ref)	1	-	-	1	-	-
**Race**						
Black African	3.11	0.93–10.39	0.066	3.85	1.06–14.03	0.041
Non-blacks (ref)	1	-	-	1	-	-
**Education**						
Primary	1.04	0.60–1.79	0.890	1.33	0.69–2.59	0.454
Secondary/tertiary (ref)	1	-	-	1	-	-
**Employment**						
Unemployed	1.72	0.76–3.89	0.193	1.26	0.69–2.31	0.197
Employed (ref)	1	-	-	1	-	-

UOR, unadjusted odd ratio; AOR, adjusted odd ratio; CI, confidence interval.

## Discussion

This study described the HIV cascade of care in an often-neglected population (people with psychiatric disorders) and addressed gaps found in the care for individuals with both conditions. The prevalence of HIV in this cohort was 18.8% and significantly higher among women (30.4% versus 7.5%). This finding highlights the critical need for strategic interventions to prevent HIV in women with psychiatric disorders in the region.

Of interest is the prevalence in this cohort, which is less than the general adult population prevalence of HIV (25.2%) in the Eastern Cape.^33^ A plausible explanation for this finding could be that this study was conducted at a regional hospital, which may have a slightly different profile of patients in comparison to the general community psychiatric population. As such, there is a need for further surveillance study at the community level to better understand the dynamics of the comorbidity of HIV and psychiatric disorders.

A lower prevalence rate was reported in Gauteng (11.0% versus 17.6%) in comparison to the general adult population, while similar prevalence rate was reported in the Western Cape Province (13.0% versus 12.6%) in comparison to the general adult population.^23,33,34^ A slightly higher prevalence rate (29.0% versus 27.0%) was reported in KZN in comparison to the general adult population.^18,33^ The difference in the prevalence rates across the various provinces could be a reflection of the HIV prevalence in the general population of the different provinces. A possible explanation for the high HIV prevalence in this cohort with co-occurring mental illness and HIV could be linked to risky sexual behaviour (unprotected sexual intercourse, multiple partners, and commercial sex work) in individuals with psychiatric disorders that has been reported in some studies.^9,35,36,37^

In this study cohort, 87.0% of the participants were aware of their HIV serostatus which is a fairly high proportion. No additional diagnosis of HIV was made after testing those who last checked their status more than 12 months prior and those who had never tested, implying a 100% awareness of status among those who were HIV positive. This finding is commendable, considering the UNAIDS global target of 90%, which was achieved at the study site. This is higher than the general population in South Africa.^33,38^ Some gaps in the current practice were evident, namely HIV testing and the re-testing after 12 months in this cohort. These gaps require further attention from clinicians working at the study site. These findings further highlight the need for inclusiveness in the care of all special populations in the global, national, and provincial agenda, irrespective of the underlying comorbid conditions.

The finding that 88% of the participants were on ART at the time of the study, was high but did not quite meet the 90% target.^38^ It is still much higher than the rate of 68% found in the general population living with HIV in South Africa who are on ART.^2^ Sixteen (26%) of the 61 participants on ART had a history of non-adherence and were re-initiated on ART. This finding corroborates previous reports which documented poor ART adherence in individuals with underlying psychiatric disorders.^25,26,27,28,29^ Similarly, there was a loss of eight patients who were not on ART, where six were previously initiated but had become non-adherent, while the remaining two were ART-naive. These findings highlight the gaps in the current practice given that these were patients attending regular follow-up clinic visits for their psychiatric disorders. Therefore, there is an urgent need for integration of care for both HIV and psychiatric disorders to improve the overall well-being of people with these comorbid diagnoses. Testing for HIV and initiation of ART for those who test positive should be part of the service provided to those accessing mental health services. Furthermore, the failure to meet the target of having 90% of people living with HIV on ART in this cohort represents a gap that must be bridged by including people with underlying psychiatric disorders in the provincial and national policy agenda, that is, through including people living with mental illness as an additional priority population in policy documents.^38^ This would bring them on par in terms of prioritisation with several other target populations such as men having sex with men, sex workers, and others are categorised as priority populations.

Also, this study reports an incredibly low virologic suppression of 53.4%, which is far from the UNAIDS global target of 90% of those on ART.^38^ This suppression rate is also far below the South African general HIV population of 87% of those who are on ART.^2^ Prior studies have also reported poor virologic response in individuals with psychiatric disorders.^39,40^ This highlights the urgency for the strengthening of HIV care in the study setting. This finding is not surprising given that poor adherence was reported by some participants in the preceding week of the study. There is a strong correlation between ART adherence and virologic suppression.^19^ Therefore, the low virologic suppression rate in this study is likely because of sub-optimal ART adherence. The finding in our study thus supports previous reports on the association of psychiatric disorders and poor adherence to ART.^29,41,42,43^ A six-fold higher viral load was found in individuals with severe psychiatric disorders and HIV in comparison with the general population.^26^ Therefore, enhanced adherence support for patients with HIV and mental illness comorbidity need to be implemented.

The finding of a five-fold higher risk of HIV infection among women with psychiatric disorders when compared to men mirrors the prevalence of HIV in the general population in South Africa.^2^ There are biological and sociodemographic explanations for the disproportionately high HIV prevalence in women when compared to men.^5,10,18,44,45^ Women are at greater risk for gender-based violence, which leads to the loss of ability to negotiate for safer sex as well as a higher risk of coercion or forced sex.^5,45,46,47^ Other factors may be a lack of education and a poorer socioeconomic status, when compared to men.^44^ Other studies have reported similar findings of a higher HIV prevalence in women with psychiatric disorders in the USA, Europe, and Asia.^10^ Also, Collins et al.,^5^ and Singh^18^ found similar results in South Africa.

The higher odds of HIV comorbidity with psychiatric disorders among black participants, after adjusting for other variables, has to be viewed in the context of the racial distribution of the study cohort and population of BCMM. It may also be reflective of previous reports which have indicated a disproportionate effect by race when it comes to HIV in the country and region.^48^ Although age, marital status, level of education, and employment status were not significantly associated with HIV comorbidity in this study, there has been a mixed relationship with these factors described in the literature. One study reported a high HIV prevalence in individuals with psychiatric disorders, who were single, attained a higher level of education, and who were employed in the USA.^8^ Another study by Henning et al.,^36^ reported a higher HIV prevalence among married individuals with psychiatric disorders. As such, the association of demographic characteristics and co-occurring conditions with HIV needs further studies.

The current study found that the majority of psychiatric disorders because of HIV in this cohort were non-affective psychosis (30%), followed by depressive disorders (13%), and lastly, bipolar disorders (6%). This could be because the participants were drawn from a clinic which offers services for people with mental health conditions who require specialist care, which may have implications for illness severity and the range of conditions seen at the clinic.

## Strength and limitations of the study

This is the first study assessing the comorbidity of HIV and psychiatric disorders in the entire Eastern Cape. The study also provides a reference epidemiological data on the treatment outcomes of HIV in this often-neglected population. As such, this study has opened doors for further studies on the dynamics of HIV disease progression in individuals with psychiatric disorders. While this study was conducted at a regional hospital, which may have a slightly different profile of patients in comparison to the general community psychiatric population, it should be noted that this is the only hospital serving the entire region. Therefore, this hospital provides community psychiatry services in addition to inpatient services for mental health disorders of varying kinds and severity. As such, this study highlights the gaps in the current care and services for people living with mental illness and HIV in the region.

Some limitations of the study include the cross-sectional design, self-reporting of awareness of HIV serostatus, and lack of information on the adherence to the current medications for the underlying psychiatric disorders. There were also no blood assays to objectively confirm the reported adherence to medication. However, evidence suggests that individuals living with HIV resort to substance abuse as a coping strategy, which has a negative correlation with medication adherence.^49^ As such, data on substance abuse in this cohort would have provided better insight into the virologic suppression in patients with HIV comorbidity. This is a notable limitation that warrants further studies in this population.

## Conclusions

This study found a high HIV prevalence rate of 18.8%, awareness rate of 87.0%, and linkage to ART of 88.4%, but a low virologic suppression of 53.4%. The prevalence of HIV was significantly associated with being female and being black, thus suggesting the need for the expansion of prevention strategies in this sub-population.

To achieve the UNAIDS global target of 90:90:90 in this sub-population, there is an urgent need for the integration of health services for HIV and psychiatric disorders. Clinicians who are involved in the care of people living with mental illnesses should be equipped to promptly identify those who need HIV testing, linkage to ART initiation, and viral load monitoring.

## References

[1] Kay ES, Batey DS, Mugavero MJ. The HIV treatment cascade and care continuum: Updates, goals, and recommendations for the future. AIDS Res Ther. 2016;13(1):35. 10.1186/s12981-016-0120-0 27826353 PMC5100316

[2] UNAIDS. UNAIDS data 2019 [homepage on the Internet]. [cited 2020 Jan 10]. Available from: https://www.unaids.org/sites/default/files/media_asset/2019-UNAIDS-data_en.pdf

[3] WHO. Consolidated guidelines on HIV testing services [homepage on the Internet]. [cited 2020 Jan 10]. Available from: https://apps.who.int/iris/bitstream/handle/10665/179870/9789241508926_eng.pdf;jsessionid=4DD5E6EB816B0A98A9189132C1605066?sequence=1

[4] NDOH. National consolidated guidelines for the prevention of mother-to-child transmission of HIV (PMTCT) and the management of HIV in children, adolescents and adults [homepage on the Internet]. [cited 2017 Jul 06]. Available from: http://www.sahivsoc.org/Files/ART%20Guidelines%2015052015.pdf

[5] Collins PY, Berkman A, Mestry K, Pillai A. HIV prevalence among men and women admitted to a South African public psychiatric hospital. AIDS Care. 2009;21(7):863–867. 10.1080/09540120802626188 20024743 PMC2800080

[6] Lommerse K, Stewart RC, Chilimba Q, Van den Akker T, Lund C. A descriptive analysis of HIV prevalence, HIV service uptake, and HIV-related risk behaviour among patients attending a mental health clinic in rural Malawi. PLoS One. 2013;8(8):e72171. 10.1371/journal.pone.0072171 24015216 PMC3756055

[7] Lundberg P, Nakasujja N, Musisi S, Thorson AE, Cantor-Graae E, Allebeck P. HIV prevalence in persons with severe mental illness in Uganda: A cross-sectional hospital-based study. Int J Ment Health Syst. 2013;7(1):20. 10.1186/1752-4458-7-20 23866085 PMC3724693

[8] Blank MB, Himelhoch SS, Balaji AB, et al. A multisite study of the prevalence of HIV with rapid testing in mental health settings. Am J Publ Health. 2014;104(12):2377–2384. 10.2105/AJPH.2013.301633 PMC4133307 24524493

[9] Guimarães MD, McKinnon K, Cournos F, et al. Correlates of HIV infection among patients with mental illness in Brazil. AIDS Care. 2014;26(4):505–513. 10.1080/09540121.2013.832722 23998905 PMC4554532

[10] Hughes E, Bassi S, Gilbody S, Bland M, Martin F. Prevalence of HIV, hepatitis B, and hepatitis C in people with severe mental illness: A systematic review and meta-analysis. The Lancet Psychiatry. 2016;3(1):40–48. 10.1016/S2215-0366(15)00357-0 26620388 PMC4703902

[11] Adams C, Zacharia S, Masters L, Coffey C, Catalan P. Mental health problems in people living with HIV: Changes in the last two decades: The London experience 1990–2014. AIDS Care. 2016;28(suppl. 1):56–59. 10.1080/09540121.2016.1146211 26888472 PMC4828597

[12] Chibanda D, Benjamin L, Weiss HA, Abas M. Mental, neurological, and substance use disorders in people living with HIV/AIDS in low-and middle-income countries. J Acquir Immune Defic Syndr. 2014;67:S54–S67. 10.1097/QAI.0000000000000258 25117961

[13] Selamawit Z, Nurilign A. Common mental disorder among HIV infected individuals at Comprehensive HIV Care and Treatment Clinic of Debre Markos referral Hospital, Ethiopia. J AIDS Clin Res. 2015;6(2):2–5.

[14] American Psychiatric Association. Fighting stigma begins at home [homepage on the Internet]. [cited 2017 Jul 06]. Available from: http://psychnews.psychiatryonline.org/doi/full/10.1176/appi.pn.2015.L1

[15] Chambers LA, Rueda S, Baker DN, et al. Stigma, HIV and health: A qualitative synthesis. BMC Public Health. 2015;15(1):848. 10.1186/s12889-015-2197-0 26334626 PMC4557823

[16] Katz IT, Ryu AE, Onuegbu AG, et al. Impact of HIV-related stigma on treatment adherence: Systematic review and meta-synthesis. J Int AIDS Soc. 2013;16(3S2):18640. 10.7448/IAS.16.3.18640 24242258 PMC3833107

[17] Joska JA, Kaliski SN, Benatar SR. Patients with severe mental illness: A new approach to testing for HIV. S Afr Med J. 2008;98(3):213–217. 18350225

[18] Singh D. Seroprevalence and HIV-associated factors among adults with severe mental illness – A vulnerable population. S Afr Med J. 2009;99(7):1–5. PMC2913716 20686643

[19] Meintjes G, Maartens G. Guidelines for antiretroviral therapy in adults. S Afr J HIV Med. 2012;13(3):114–133. 10.4102/sajhivmed.v13i3.125 PMC7564911 33101723

[20] Yehia BR, Cui W, Thompson WW, et al. HIV testing among adults with mental illness in the United States. AIDS Patient Care STDs. 2014;28(12):628–634. 10.1089/apc.2014.0196 25459230 PMC4250950

[21] Bauer-Staeb C, Jörgensen L, Lewis G, Dalman C, Osborn DP, Hayes JF. Prevalence and risk factors for HIV, hepatitis B, and hepatitis C in people with severe mental illness: A total population study of Sweden. The Lancet Psychiatry. 2017;4(9):685–693. 10.1016/S2215-0366(17)30253-5 28687481 PMC5573766

[22] Breuer E, Myer L, Struthers H, Joska JA. HIV/AIDS and mental health research in sub-Saharan Africa: A systematic review. Afr J AIDS Res. 2011;10(2):101–122. 10.2989/16085906.2011.593373 25859733

[23] Franken H, Parker J, Allen R, Wicomb RA. A profile of adult acute admissions to Lentegeur Psychiatric Hospital, South Africa. S Afr J Psychiatr. 2019;25(1):1–7. 10.4102/sajpsychiatry.v25i0.1244 PMC6779967 31616578

[24] Mere SM, Paruk S. Chart review of human immunodeficiency virus status in patients admitted with psychosis in Durban, South Africa. S Afr J Psychiatry. 2018;24(1):a1129. 10.4102/sajpsychiatry.v24i0.1129 PMC6138071 30263214

[25] Moore DJ, Posada C. HIV and psychiatric co-morbidities: What do we know and what can we do. Psychology and AIDS Exchange Newsletter. New York, NY: American Psychological Association, 2013; p. 1.

[26] Blank MB, Himelhoch S, Walkup J, Eisenberg MM. Treatment considerations for HIV-infected individuals with severe mental illness. Current HIV/AIDS Rep. 2013;10(4):371–379. 10.1007/s11904-013-0179-3 PMC3857330 24158425

[27] Chander G, Himelhoch S, Moore RD. Substance abuse and psychiatric disorders in HIV-positive patients. Drugs. 2006;66(6):769–789. 10.2165/00003495-200666060-00004 16706551

[28] Thom R, Freeman M. Serious mental illness and HIV/AIDS. S Afr J Psychiatr. 2006;12(1):4–8. 10.4102/sajpsychiatry.v12i1.45

[29] Springer SA, Dushaj A, Azar MM. The impact of DSM-IV mental disorders on adherence to combination antiretroviral therapy among adult persons living with HIV/AIDS: A systematic review. AIDS Behav. 2012;16(8):2119–2143. 10.1007/s10461-012-0212-3 22644066 PMC3481055

[30] Uys H. Prevalence and clinical presentation of HIV positive female psychiatric inpatients. Afr J Psychiatr. 2013;16(1):23–28. 10.4314/ajpsy.v16i1.4 23417632

[31] Census. Census 2011 [homepage on the Internet]. [cited 2017 Jun 29]. Available from: https://census2011.adrianfrith.com/place/2

[32] Hansoti B, Mwinnyaa G, Hahn E, et al. Targeting the HIV epidemic in South Africa: The need for testing and linkage to care in emergency departments. EClinicalMedicine. 2019;15:14–22. 10.1016/j.eclinm.2019.08.007 31709410 PMC6833451

[33] Human Sciences Research Council. The fifth South African national HIV prevalence, incidence, behaviour and communication survey, 2017: HIV impact assessment summary report [homepage on the Internet]. [cited 2020 Jan 10]. Available from: http://www.hsrc.ac.za/uploads/pageContent/9234/SABSSMV_Impact_Assessment_Summary_ZA_ADS_cleared_PDFA4.pdf

[34] Henning MP, Krüger C, Fletcher L. HIV sero-positivity in recently admitted and long-term psychiatric in-patients: Prevalence and diagnostic profile. Afr J Psychiatr. 2012;15(1):47–53. 10.4314/ajpsy.v15i1.7 22344763

[35] Tucker JS, Kanouse DE, Miu A, Koegel P, Sullivan G. HIV risk behaviors and their correlates among HIV-positive adults with serious mental illness. AIDS Behav. 2003;7(1):29–40. 10.1023/A:1022557222690 14534388

[36] Carey MP, Carey KB, Maisto SA, Gordon CM, Vanable PA. Prevalence and correlates of sexual activity and HIV-related risk behavior among psychiatric outpatients. J Consult Clin Psychol. 2001;69(5):846. 10.1037/0022-006X.69.5.846 11680563 PMC2424203

[37] Obo CS, Sori LM, Abegaz TM, Molla BT. Risky sexual behavior and associated factors among patients with bipolar disorders in Ethiopia. BMC Psychiatry. 2019;19(1):313. 10.1186/s12888-019-2313-2 31653241 PMC6815011

[38] UNAIDS. UNAIDS 2016–2021 strategy, on the fast-track to end AIDS [homepage on the Internet]. [cited 2017 Jun 21]. Available from: https://www.unaids.org/sites/default/files/media_asset/20151027_UNAIDS_PCB37_15_18_EN_rev1.pdf

[39] Coviello DM, Lovato R, Apostol K, et al. Prevalence of HIV viral load suppression among psychiatric inpatients with comorbid substance use disorders. Community Ment Health J. 2018;54(8):1146–1153. 10.1007/s10597-018-0284-2 29752639 PMC6230497

[40] Pence BW, Miller WC, Gaynes BN, Eron Jr JJ. Psychiatric illness and virologic response in patients initiating highly active antiretroviral therapy. J Acquir Immune Defic Syndr. 2007;44(2):159–166. 10.1097/QAI.0b013e31802c2f51 17146374

[41] Mellins CA, Havens JF, McCaskill EO, Leu CS, Brudney K, Chesney MA. Mental health, substance use and disclosure are significantly associated with the medical treatment adherence of HIV-infected mothers. Psychol Health Med. 2002;7(4):451–460. 10.1080/1354850021000015267

[42] Mellins CA, Havens JF, McDonnell C, et al. Adherence to antiretroviral medications and medical care in HIV-infected adults diagnosed with mental and substance abuse disorders. AIDS Care. 2009;21(2):168–177. 10.1080/09540120802001705 19229685 PMC5584780

[43] Uldall KK, Palmer NB, Whetten K, Mellins C, For The HIV/AIDS Treatment Adherence, Health Outcomes and Cost Study Group. Adherence in people living with HIV/AIDS, mental illness, and chemical dependency: A review of the literature. AIDS Care. 2004;16(suppl. 1):71–96. 10.1080/09540120412331315277 15736823

[44] Magadi MA. Understanding the gender disparity in HIV infection across countries in sub-Saharan Africa: Evidence from the Demographic and Health Surveys. Sociol Health Illness. 2011;33(4):522–539. 10.1111/j.1467-9566.2010.01304.x PMC3412216 21545443

[45] Evans M, Risher K, Zungu N, et al. Age-disparate sex and HIV risk for young women from 2002 to 2012 in South Africa. J Int AIDS Soc. 2016;19(1):21310. 10.7448/IAS.19.1.21310 28364564 PMC5384594

[46] Howard LM, Trevillion K, Khalifeh H, Woodall A, Agnew-Davies R, Feder G. Domestic violence and severe psychiatric disorders: Prevalence and interventions. Psychol Med. 2010;40(6):881–893. 10.1017/S0033291709991589 19891808

[47] Atteraya MS, Kimm H, Song IH. Women’s autonomy in negotiating safer sex to prevent HIV: Findings from the 2011 Nepal demographic and health survey. AIDS Educ Prev. 2014;26(1):1–2. 10.1521/aeap.2014.26.1.1 24450274

[48] UNAIDS. UNAIDS data 2019 [homepage on the Internet]. [cited 2018 Feb 28]. Available from: https://www.unaids.org/sites/default/files/media_asset/2017_data-book_en.pdf

[49] Gonzalez A, Mimiaga MJ, Israel J, Bedoya CA, Safren SA. Substance use predictors of poor medication adherence: The role of substance use coping among HIV-infected patients in opioid dependence treatment. AIDS Behav. 2013;17(1):168–173. 10.1007/s10461-012-0319-6 23008124 PMC3632258

